# Supplementary material for: EMP2 Serves as a Functional Biomarker for Chemotherapy-Resistant Triple-Negative Breast Cancer
Source: Cancers (Basel). 2024 Apr 12;16(8):1481. doi: 10.3390/cancers16081481 (PMC11048488; doi:10.3390/cancers16081481)
Supplement: Supplementary file 1 [file cancers-16-01481-s001.zip › supplemental figures.pdf]

Figure S1

Electropherogram
Lot: 4640-848888 Reducing

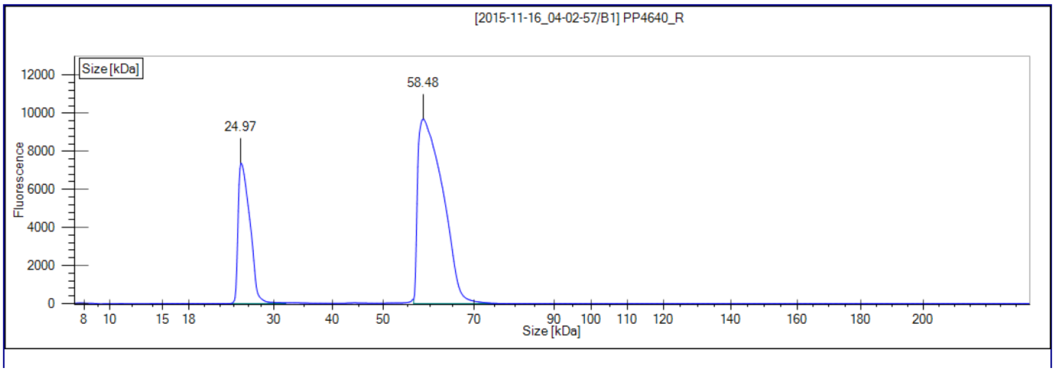

Figure S1. Electropherogram of the anti-EMP2 mAb. Image shows the reduced IgG1 antibody.

Figure S2

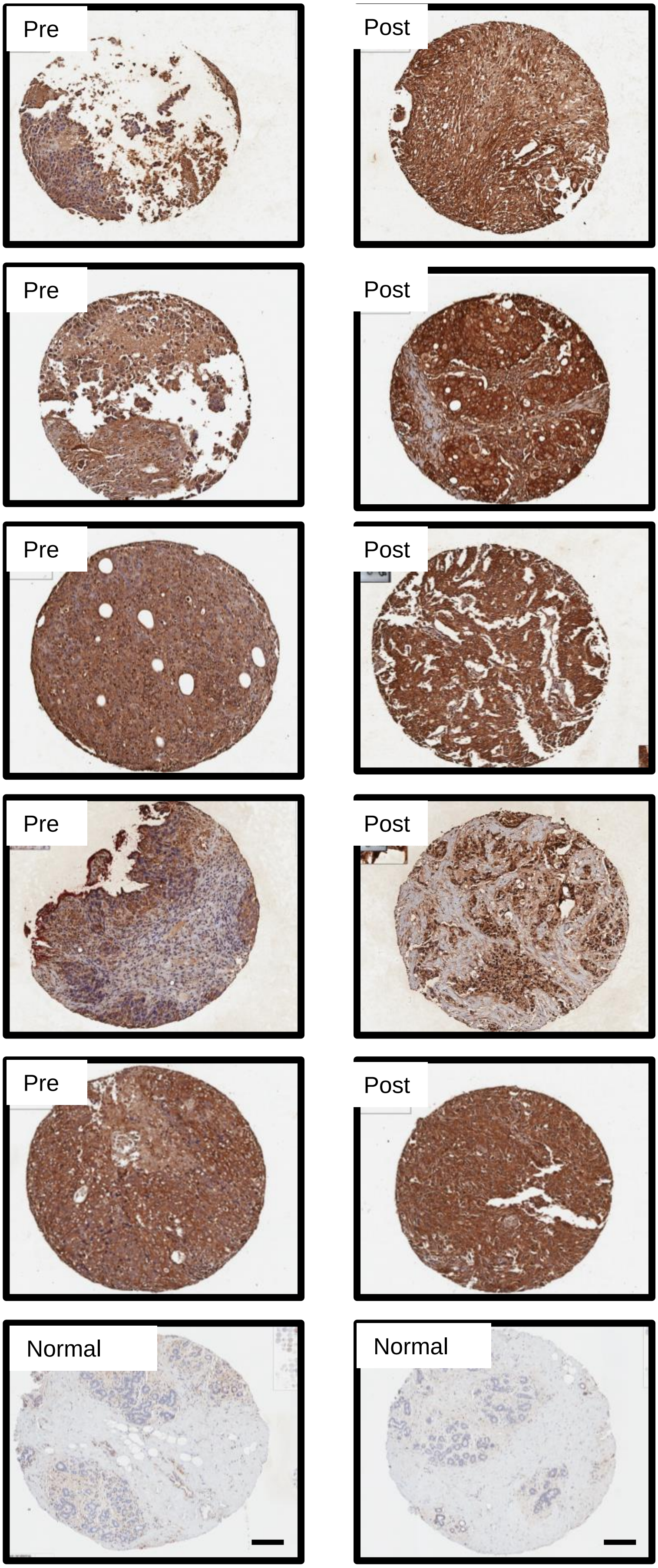

Figure S2. EMP2 staining in patient tumors pre and post neoadjuvant chemotherapy. Representative naïve or post- neoadjuvant, taxane-based chemotherapy images are shown. Normal breast samples are provided as a control from a parallel TMA stained at the same time. Scalebar = 100μm.

Figure S3

A

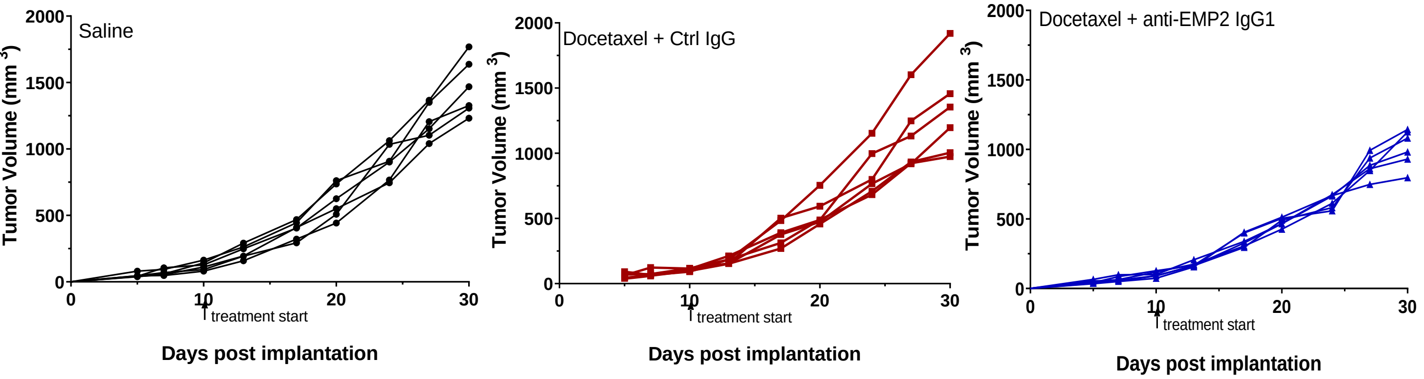

Saline
Docetaxel + Ctrl IgG
Docetaxel + anti-EMP2 IgG1
Tumor Volume (mm 3)
2000
1500
1000
500
0
0
10
20
30
treatment start
Days post implantation

B

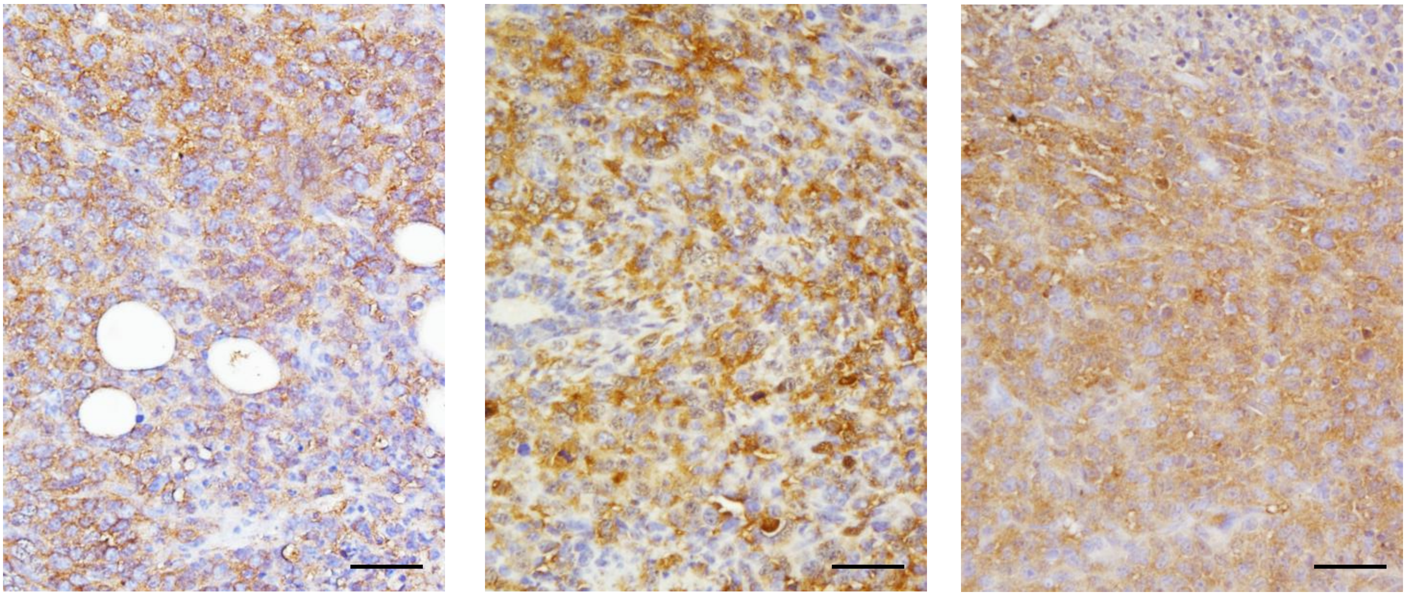

Figure S3. Treatment with anti-EMP2 mAbs reduces tumor load in taxane resistant 4T1 tumors. (**A**) 4T1 tumors were implanted into female Balb/c mice. Tumors were treated twice a week with control or anti-EMP2 mAbs in combination with systemic weekly docetaxel injections. Individual tumor growth per animals is shown, with grouped data shown in Figure 4B. (**B**) EMP2 expression was evaluated in naïve and post treatment tumors by immunohistochemistry, with representative images shown. Treatment with anti-EMP2 mAbs reduces EMP2 levels in taxane resistant 4T1 tumors. Scalebar = 50μm.

Figure S4

A

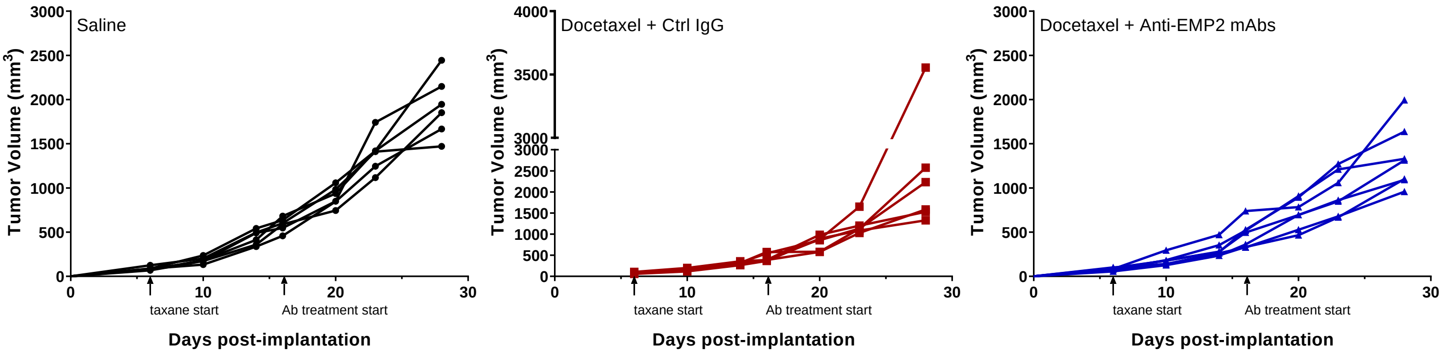

Saline
Docetaxel + Ctrl IgG
Docetaxel + Anti-EMP2 mAbs
Tumor Volume (mm^3)
taxane start
Ab treatment start
Days post-implantation

B

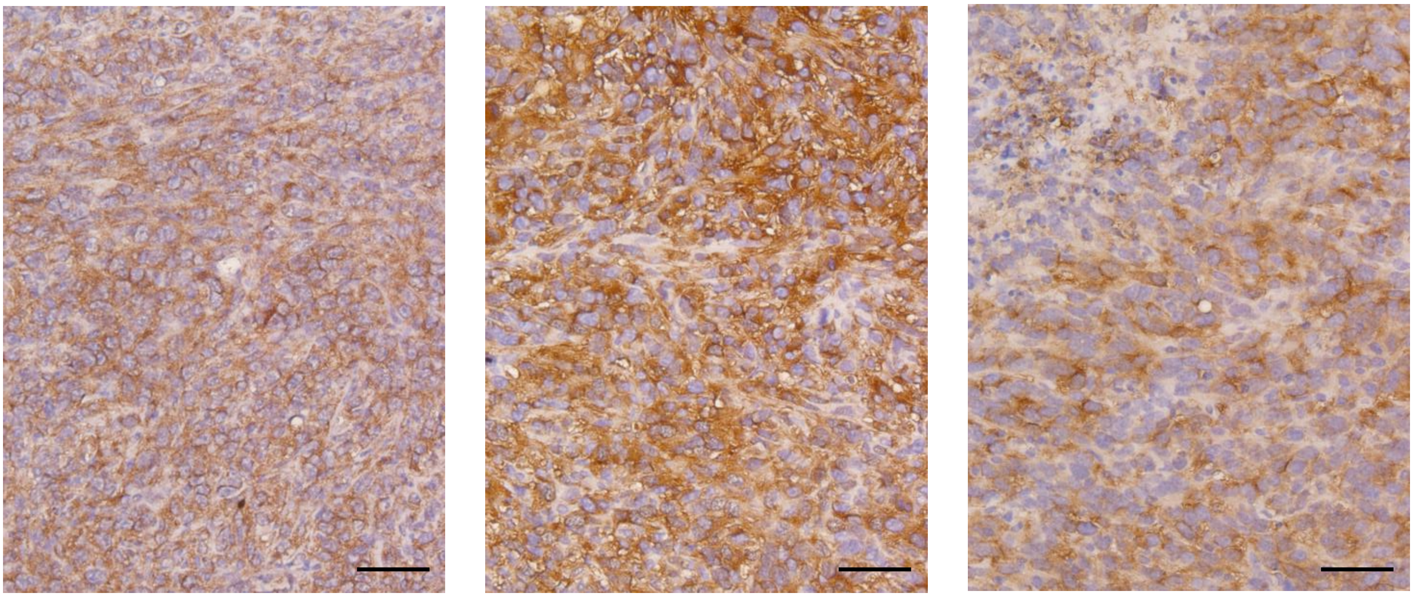

Figure S4. Treatment with anti-EMP2 mAbs reduces tumor load in taxane resistant 4T1 tumors. (**A**) 4T1 tumors were implanted into female Balb/c mice. Tumors were treated weekly with systemic docetaxel. After 2 cycles of chemotherapy, control or anti-EMP2 mAb treatments were added to the regimen. Individual tumor growth per animals is shown, and grouped data is presented in Figure 4D. (**B**) EMP2 expression was evaluated in naïve and post treatment tumors, with representative images shown. Scalebar = 50μm.
