# Supplementary material for: EMP2 Serves as a Functional Biomarker for Chemotherapy-Resistant Triple-Negative Breast Cancer
Source: Cancers (Basel). 2024 Apr 12;16(8):1481. doi: 10.3390/cancers16081481 (PMC11048488; doi:10.3390/cancers16081481)
Supplement: Supplementary file 1 [file cancers-16-01481-s001.zip › Supplemental Methods_Final.pdf]

Supplemental Methods

Tumor disassociation

Taxane resistant MDA-MB-231 tumors were disassociated using the MACS human Tumor Dissociation Kit (Miltenyi Biotec, San Diego, CA) as per the manufacturer's specifications. Cells were then washed in saline and filtered using a 40 micron nylon mesh. To remove red blood cells, the suspension was incubated in ACK Lysing buffer for 3 min at RT and then washed in PBS prior to utilization in the cellular proliferation or mammosphere assays.

Cellular proliferation

5000 taxane resistant cells isolated from tumors were plated in a black, clear-bottom 96-well plate (Corning, Glendale, AZ) and treated at the time of plating with PBS, control mAbs (human IgG; Sigma Aldrich, I4506) or anti-EMP2 mAbs at the concentrations indicated within the figure legend. Cellular proliferation was measured using the CellTiter-Glo Luminescent Cell Viability Assay as per manufacturer's instructions (Promega, Madison, WI) using the GloMax-Multi Base Instrument (Promega, #E7031) after 5-7 days.

Mammosphere assay

A single cell suspension of taxane resistant cells was prepared as above, with a total of 1000 cells/cm^2 plated on 1.2% polyHEMA (Poly (2-hydroxyethylmethacrylate) coated tissue culture plates. Cells were grown in Mammocult media supplemented with 1% v/v L-glutamine, 1% v/v Penicillin-Streptomycin, 1% sodium pyruvate, 4 μg/ml heparin (StemCell Technology, Seattle, WA), 0.48 µg/mL hydrocortisone (Millipore Sigma, St. Louis, MO), and 10% v/v MammoCult proliferation supplement (StemCell Technology) for two weeks. Treatment of tumorspheres with control or anti-EMP2 mAbs was done at time of plating in triplicate at the concentrations indicated in the figures.

Immunohistochemistry

Immunohistochemical staining was performed on paraffin embedded, formalin fixed 5mm sections. Sections were deparaffinized in xylene and rehydrated using graded alcohols. For antigen retrieval, slides were immersed in 0.01 M citrate buffer, pH 6.0 at 95°C for 20 minutes. Samples were blocked in DAKO Serum-Free Protein Block for 20 minutes and subsequently incubated in primary antibody overnight at 4°C. For EMP2 staining, the sections were incubated with rabbit anti-mouse EMP2 antisera (1:500), anti-human EMP2 antisera (1:500), or control rabbit serum at the same dilutions overnight, as previously described[1, 2]. Bound anti-EMP2 immunoglobulins were visualized using the DAKO Envision rabbit kit according to manufacturer's instructions (Agilent, Santa Clara, CA). EMP2 expression was detected using 3,3'-diaminobenzidine (DAB) substrate, and the nuclei were counterstained using diluted Harris Modified Method Hematoxylin (Vector Labs, Burlingame, CA). Samples were scored by both the intensity of staining and the percentage of neoplastic cells expressing the antigen. Scores for negative staining were 0, weak positive 1.0-2.0 and strongly positive between 2-3.

In vivo targeting of syngeneic models

Animal studies were performed in strict accordance with the recommendations in the Guide for the Care and Use of Laboratory Animals of the National Institutes of Health. The Animal Research Committee at the University of California, Los Angeles approved all procedures. All

efforts were made to minimize animal suffering. To create MDA-MB-231 models, $4x10^6$ wildtype cells were mixed 1:1 in Matrigel Matrix (Corning, # 354234) and implanted into the third mammary fat pad of Balb/c Nude mice. Docetaxel (Millipore Sigma) was solubilized in DMSO at a concentration of 5 mg/ml solution in PBS containing 0.5% DMSO [14]. When the tumors reached between 50-80 mm^3, mice were randomly assigned to four groups with 4 mice per group, and they were treated via intraperitoneal injection (IP) with either control mAbs (Human IgG), both docetaxel (20 mg/kg, weekly) and control mAbs (10mg/kg, twice a week), single agent anti-EMP2 mAbs (10 mg/kg, twice a week), or both docetaxel and anti-EMP2 mAbs. Docetaxel and antibody treatments were not given on the same day to minimize the volume of reagents given IP to an individual animal.

To create 4T1/FLuc models, $5x10^4$ cells were mixed 1:1 in Geltrex (Thermofisher) and injected into the third mammary fat pad of Balb/c mice. Pharmaceutical grade docetaxel was obtained as a 10mg/ml pre-resuspended ampule (Sandoz #6675805003). In the first experiment, when tumors reached $100mm^3$, they were treated with saline or concurrently with docetaxel (10 mg/kg, weekly) and control mAbs or docetaxel and anti-EMP2 mAbs. In a separate experiment, animals were treated sequentially with docetaxel first (to create resistant cells) followed by the addition of control or anti-EMP2 mAbs when tumors approached $500mm^3$. The number of animals used per group is indicated in the figure legend. In all experiments, tumor volumes were measured twice a week and calculated according to the formula Volume=LengthxWidth (smaller side)^2/2.

Database mining

To assess transcriptomic EMP2 expression in relation to clinical characteristics of treatment responsiveness and survival, respectively, the open-access biomarker evaluation tools ROC Plotter [3] and KM Plotter [4] were used. We used ROC Plotter to stratify samples from all breast cancer patients receiving taxane-based chemotherapy into two cohorts based on achievement of a pathological complete response. Using KM Plotter, we performed multiple queries on Affy ID: 225078_at. We separated all BC patients or TNBC patients (ER-, PR-, and HER2-) who received any systemic chemotherapy into high and low EMP2-expression cohorts, with the threshold of optimal performance automatically computed by KM Plotter.

Statistical analysis

All statistical analyses described were conducted using R or GraphPad Prism software (version 5, GraphPad Software Inc., La Jolla, CA), and normality tested between groups using the Shapiro-Wilk normality test. Kaplan–Meier recurrence or overall survival analysis was carried out using the dependent variables progression-free survival and overall survival stratified by high and low EMP2 histological score from both the clinical cohort and TCGA cohort. Unpaired and paired t-tests were used to evaluate differences in EMP2 before and after taxane treatment. Errors are presented as the standard error of the mean (SEM) or standard deviation (SD) as indicated in the figure legends. $p<0.05$ was considered statistically significant and $p<0.1$ was considered a statistical trend. Specific tests performed are indicated within the manuscript or figure legends.

Data and code availability

This paper analyzes some existing publicly available data via open access sites. This paper does not report original code. Any additional information required to reanalyze the data reported in this paper is available from the lead contact upon request.

REFERENCES

1. Wadehra M, Sulur GG, Braun J, Gordon LK, Goodglick L: **Epithelial membrane protein-2 is expressed in discrete anatomical regions of the eye**. *Exp Mol Pathol* 2003, **74**(2):106-112.
2. Wang CX, Wadehra M, Fisk BC, Goodglick L, Braun J: **Epithelial membrane protein 2, a 4-transmembrane protein that suppresses B-cell lymphoma tumorigenicity**. *Blood* 2001, **97**(12):3890-3895.
3. Fekete JT, Győrffy B: **ROCplot.org: Validating predictive biomarkers of chemotherapy/hormonal therapy/anti-HER2 therapy using transcriptomic data of 3,104 breast cancer patients**. *Int J Cancer* 2019, **145**(11):3140-3151.
4. Gyorffy B, Lanczky A, Szallasi Z: **Implementing an online tool for genome-wide validation of survival-associated biomarkers in ovarian-cancer using microarray data from 1287 patients**. *Endocr Relat Cancer* 2012, **19**(2):197-208.
