# Supplementary material for: EMP2 Serves as a Functional Biomarker for Chemotherapy-Resistant Triple-Negative Breast Cancer
Source: Cancers (Basel). 2024 Apr 12;16(8):1481. doi: 10.3390/cancers16081481 (PMC11048488; doi:10.3390/cancers16081481)
Supplement: Supplementary file 1 [file cancers-16-01481-s001.zip › Table S1.pdf]

Table S1

Characteristics	EMP2 increased after chemotherapy (yes/no)	p-value
**Age**		
median	53 yrs	0.93§§
range	26-72 yrs	
**Ethnicity**		
Caucasian	4	
Hispanic	1	1§
African American	2	
Asian	1	
**Stage**		
II	6	0.46 §
III/IV	2	
**Lymph node spread**		
present	5	0.25 §
absent	3	
**Menopausal status**		
pre-menopausal	4	0.43 §
post-menopausal	4	
**Recurrence**		
Yes	6	
No	2	0.036 §

§§ Spearman correlation
§ Fisher exact test
